# Nutritional and performance effects of shrimp meal and yam bean as sustainable ingredients in laying hen diets

**DOI:** 10.5713/ab.250559

**Published:** 2025-12-18

**Authors:** Nonthiwat Taesuk, Wiriya Thongsomboon, Phatthanawan Kaeowiset, Hathaipan Kaenjak, Anchalee Namsri, Anut Chantiratikul, Doungnapa Promket, Manisa Sangkaew

**Affiliations:** 1 Department of Biology, Faculty of Science, Mahasarakham University, Maha Sarakham, Thailand; 2 Sustainable Approaches for Materials, Agriculture, and Health Technology (SAMAHT) Research Unit, Mahasarakham University, Maha Sarakham, Thailand; 3 Department of Chemistry, Faculty of Science, Mahasarakham University, Maha Sarakham, Thailand; 4 Major of Animal Science, Department of Agricultural Technology, Faculty of Technology, Mahasarakham University, Maha Sarakham, Thailand

**Keywords:** Alternative Feed Sources, Digestibility, Egg Quality, Laying Hens, Laying Performance, Nitrogen Retention

## Abstract

**Objective:**

Rising poultry feed costs and shortages in Southeast Asia, particularly in Thailand, underscore the need for sustainable alternatives. This study evaluated shrimp meal (SM), a protein-rich by-product, and yam bean (YB), an underutilized energy-rich root crop, as promising alternative feed ingredients for laying hens.

**Methods:**

An *in vitro* analysis was conducted to determine the chemical composition and digestibility of diets containing SM and varying levels of YB. An 8-week *in vivo* trial was conducted using 90 ISA-Brown hens (25-week-old), divided into nine dietary groups: one control and eight treatment groups with 10% or 15% SM combined with 0%, 3%, 6%, and 9% YB. Laying performance, egg quality and composition, and nitrogen (N) retention were assessed.

**Results:**

SM was rich in protein while YB contained high N-free extracts and gross energy, supporting their use as alternative protein and energy sources, respectively. The inclusion of SM and YB had no negative effect on *in vitro* dry matter or protein digestibility. *In vivo*, combined inclusion of up to 15% SM and 9% YB did not adversely affect laying performance, egg quality, nutrient composition, or N retention compared to the control. A main effect analysis revealed that SM enhanced yolk color, eggshell weight, and eggshell thickness (p<0.05), while reducing egg fat content (p<0.05). Although higher levels of SM were independently associated with reduced hen-day egg production and egg mass, these effects were mitigated when SM was combined with YB, resulting in no differences from the control group at the highest inclusion levels (15% SM and 9% YB).

**Conclusion:**

The SM and YB are viable, eco-friendly alternatives to conventional poultry feed ingredients. Their combined inclusion, up to 15% SM and 9% YB, is recommended to maintain laying performance and egg quality while promoting sustainable feeding practices through the utilization of processing by-product and underutilized local crops.

## INTRODUCTION

The persistent shortage and rising costs of livestock feed, particularly in poultry production, have posed substantial challenges for both smallholder and commercial farmers in recent decades. This issue is especially severe in Southeast Asian countries such as Thailand, a major producer and exporter of poultry meat and eggs. The increasing cost of conventional feed ingredients, particularly maize and soybean meal, which serve as the primary energy and protein sources, raises significant concerns about the sustainability and profitability of poultry production, especially in developing countries. Feed alone accounts for approximately 70% of the total production cost in poultry farming [1], underscoring the urgent need to identify alternative, cost-effective, and locally available feed resources to enhance feed security and reduce production expenses.

Among poultry feed components, energy sources represent the largest proportion, with maize typically comprising 60 to 70% of formulated diets [2]. However, maize availability is often subject to market fluctuations and periodic shortages, largely due to reliance on imports to offset inadequate domestic production. This has driven the search for alternative energy-rich ingredients. One promising substitute is yam bean (YB; *Pachyrhizus erosus*), a tropical leguminous root crop characterized by high carbohydrate content [3] and low levels of anti-nutritional factors [4], making it a suitable energy source in poultry diets. In addition to its nutritional value, YB tubers contain bioactive compounds such as flavonoids and inulin [5], which may offer functional benefits by supporting animal health and performance. Flavonoids are well known for their antioxidant properties [6], while inulin is widely recognized as a prebiotic that enhances gut health in chickens [7]. Notably, YB is a resilient crop capable of thriving in harsh environments and is widely cultivated yet underutilized in Thailand, particularly in the northeastern region, making it a promising local alternative to maize that could strengthen feed security for poultry producers.

Shrimp meal (SM), a by-product of the shrimp processing industry, is another potential feed ingredient, valued for its high protein content and amino acid profile comparable to soybean meal [8]. Previous studies have explored the inclusion of SM at levels ranging from 5% to 25% in laying hen diets, though results have been inconsistent across studies [9–11]. In addition to its nutritional properties, SM contains functional components such as astaxanthin and chitin [12]. Astaxanthin has been reported to exhibit strong antioxidant activity [13] and enhance pigmentation in aquaculture species [14,15] as well as in egg yolk of laying hens [16]. Although chitin is often considered as an antinutritional factor due to its impact on nutrient digestibility [17], low inclusion levels have been associated with reduced cholesterol and triglyceride concentrations in chickens [18]. Given its potential nutritional and functional benefits, SM holds promise as an alternative protein source for improving both performance and egg quality in laying hens.

Integrating YB and SM into poultry diets presents an eco-friendly and sustainable strategy for reducing dependence on conventional ingredients. While several studies have investigated the use of SM in poultry nutrition, the findings remain variable, and research on YB as an energy source is still limited. More importantly, the combined effects of SM and YB in the diets of laying hens have not been extensively studied. To address this gap, the present study comprised two trials. First, due to the limited information on the use of YB as a feed ingredient, an *in vitro* trial was conducted to evaluate its effect on the digestibility of an SM-based diet. Second, to determine whether the combination of SM and YB could serve as alternative feed ingredients in laying hen diets, an *in vivo* study was carried out to assess their effects on productive performance, egg quality, egg nutrient composition, nitrogen (N) retention, and nutrient digestibility.

## MATERIALS AND METHODS

### Experiment 1: *in vitro* study

#### Yam bean and shrimp meal preparation and chemical analysis

YB (*P. erosus*) tubers, harvested after three months of cultivation, were commercially obtained from local farms in Burabue Subdistrict, Maha Sarakham Province, Thailand. The tubers were thoroughly washed with tap water, peeled, and thinly sliced. The slices were then dried at 50°C until the moisture content fell below 10%. After drying, the slices were ground to pass through a 1.0-mm mesh screen and stored at room temperature for subsequent chemical analysis.

For SM preparation, a dried by-product of shrimp processing, including heads and shells of White Leg shrimps (*Litopenaeus vannamei*), was commercially purchased from a local distributor in Samut Songkhram Province, Thailand. The dried SM was ground to pass through a 1.0-mm mesh screen for further chemical analysis.

The chemical compositions of both YB and SM, including crude protein (CP), ether extract, crude fiber, ash, and N-free extract (NFE), were determined following the standard methods of the AOAC [19] (Table 1). The gross energy values were measured using a C3000 isoperibol bomb calorimeter (IKA). Chitin content in SM was measured according to the method described by Ghanem et al [20].

#### Experimental diets and in vitro digestibility assay

To evaluate the effects of YB on the *in vitro* digestibility of laying hen diets containing SM, five experimental diets were formulated: one control diet and four diets containing 10% SM supplemented with 0%, 3%, 6%, and 9% YB, replacing equivalent amounts of maize (Table 2). All diets were formulated to meet the nutrient requirements for laying hens as recommended by the NRC [21].

The *in vitro* digestibility of YB, SM, and the experimental diets was assessed using a modified method based on Saunders et al [22], adapted to simulate poultry physiological conditions. Briefly, 250 mg of each diet sample was suspended in 15 mL of 0.1 *N* HCl containing pepsin (>10,000 NFU/mg; HiMedia Laboratories) and incubated at 41°C for 3 hours. Afterward, the mixture was neutralized with 0.5 *N* NaOH and combined with phosphate buffer (pH 8.0) containing pancreatin (>100 USP U/mg amylase, >100 USP U/mg protease, >8 USP U/mg lipase; HiMedia Laboratories), then shaken at 41°C for 24 hours. The resulting mixture was filtered, rinsed with distilled water, and dried. The dry matter (DM) and CP contents of the original samples and the digested residues were analyzed to determine *in vitro* DM digestibility (IVDMD) and *in vitro* CP digestibility (IVCPD).

### Experiment 2: *in vivo* study

#### Dietary treatments and husbandry

Ninety ISA-Brown laying hens, 25 weeks of age, were randomly allocated to nine treatment groups, with ten hens per group and an average initial laying rate of 98%. The hens were housed individually in cages under a 16L:8D light regime. The birds were assigned to one of nine dietary treatments, including a control diet without SM or YB, and eight experimental diets arranged in a 2×4 factorial design: two levels of SM (10% and 15%) combined with four levels of YB (0%, 3%, 6%, and 9%; Table 3). The SM and YB were incorporated into the diets at the expense of soybean meal and maize, respectively. All diets were formulated to meet or slightly exceed the nutrient requirements of laying hens as recommended by the ISA Brown commercial management guide [23]. Chitin-derived N from SM was excluded from the CP calculations, as there is no clear evidence that poultry can utilize this form of N. The experimental feeding period lasted eight weeks, with the first week designated for adaptation and the following seven weeks for data collection. Feed was provided at approximately 120 g/hen/day, while water was available *ad libitum*.

#### Laying performance, egg quality, and nutrient compositions in eggs

Egg production and feed intake (FI) were recorded daily. Hen-day egg production (HDEP) was calculated on a hen per day basis. Egg mass was calculated from egg production and egg weight, using the following equation: egg mass (g/hen) = (egg production × egg weight) / period (day). Feed conversion ratio (FCR) was determined as the ratio of feed consumed to egg mass, and changes in body weight (BW) were calculated as the difference between the initial and final BW.

Each week, five eggs were randomly selected from each group for egg quality measurement. The Haugh unit (HU) was measured using an egg freshness meter (TSS, version 2.1 – release 27E7). Yolk diameter was measured using a Vernier caliper (Insize 6-inch/150 mm Digital Caliper), and yolk weight was recorded. Yolk color was visually assessed using the standard La Roche scale (Roche Yolk Color Fan, Bröring Technology). Eggshells were weighed after drying at 100°C for 2 hours, and shell thickness was measured using a micrometer (IP65 Micrometer; Mitutoyo).

During the final three days of the experimental period, three eggs were randomly selected from each group for nutrient composition analysis. The eggs were broken, pooled within each group, and dried at 70°C for 24 hours. The dried samples were ground to pass through a 1.0-mm screen and then analyzed for chemical composition.

#### Nutrient digestibility and nitrogen balance

During the final three days of the experimental period, three birds per group were randomly selected. Excreta from each bird were quantitatively collected twice daily over the three-day period and pooled to form one composite sample per bird, ensuring that all samples were free from contamination by feathers or feed. Samples were stored at −20°C until further analysis. Prior to analysis, frozen excreta were thawed, dried in a hot-air oven at 60°C for 24 hours, and finely ground to pass through a 1.0-mm mesh screen. The processed samples were then analyzed for chemical composition using the standard AOAC method [19].

During the same period as excreta collection, three eggs were collected over a 72-hour interval from the same birds selected for excreta sampling to determine egg N content. The eggs were weighed, broken, and the albumen and yolk were pooled per bird. The pooled mixture was weighed, and a 4 mL aliquot was taken for DM determination by drying at 70°C for 24 hours. The remaining sample was dried and subsequently analyzed for N content. Nutrient digestibility and N balance, including intake, excretion, and retention as total, egg, or body N, were calculated based on the method described by Roberts et al [24]. All values were expressed on a DM basis per hen per day.

### Calculation and statistical analysis

The cost of feeding per kilogram of egg production for laying hens under each dietary treatment was calculated using the formula provided by Lokaewmanee et al [25]: Cost of feeding per kilogram of egg production (baht/kg of egg) = FCR×price of 1 kg of feed. The present study was conducted using a 2×4+1 augmented factorial treatment arrangement, which included one control group and two levels of SM in combination with four levels of YB. Statistical analyses were conducted using R software ver. 4.3.1 [26]. For normally distributed data, a two-way analysis of variance (ANOVA) was used to assess the effects of main and combined factors. When the data did not meet the assumption of normality, the Kruskal–Wallis test was applied as a non-parametric alternative. Statistical significance was determined at p<0.05. Groups with significant differences were denoted by different superscript letters. Post-hoc comparisons were performed using Tukey’s HSD test following ANOVA, and Dunn’s test was used after the Kruskal–Wallis test to identify pairwise differences.

## RESULTS

### Experiment 1: *in vitro* study

#### Chemical compositions and in vitro digestibility in shrimp meal and yam bean

The SM mainly consisted of protein, accounted for approximately 47.7% of its content, followed by ash at 24.9% (Table 1). It contained low levels of fat and NFE, while its gross energy content was relatively high, at approximately 3,804 kcal/kg. The crude fiber content of SM was approximately 11%, which closely corresponded to its chitin content, measured at 14.1%. In contrast, YB was primarily composed of NFE (approximately 88.1%) and contained only small amounts of other nutrients, while also exhibiting a high gross energy content of about 3,603 kcal/kg.

Regarding *in vitro* digestibility, SM showed a low IVDMD of approximately 47.8%, but a relatively high IVCPD of around 73.5%. Conversely, YB exhibited a high IVDMD value of about 72.3%, but a low IVCPD of approximately 40.5%.

#### In vitro digestibility of diets containing shrimp meal with varying levels of yam bean

**Figure 1:**
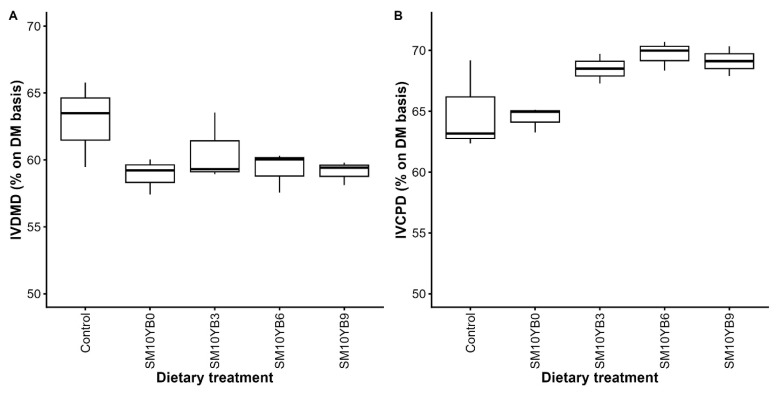
Effects of shrimp meal and yam bean inclusion on *in vitro* digestibility of dry matter (A) and crude protein (B). The diets included a control diet and diets containing 10% SM with varying levels of YB at 0%, 3%, 6%, and 9%. Data represent mean±SD (n = 3). SM, shrimp meal; YB, yam bean; IVDMD, *in vitro* dry matter digestibility; IVCPD, in vitro crude protein digestibility; SD, standard deviation.

The control diet showed IVDMD and IVCPD values of approximately 62.9% and 64.9%, respectively. These values were not different from those observed in diets containing 10% SM. Furthermore, the inclusion of YB in the SM-based diets did not adversely affect (p<0.05) either IVDMD or IVCPD, even at inclusion levels of up to 9%, when compared with the control or SM-only diets, as shown in Figure 1 and Supplement 1.

### Experiment 2: *in vivo* study

#### Laying performance and cost of feeding

Laying hens fed the control diet exhibited average performance, with HDEP at 85%, FI at 110 g/bird/day, egg weight at 60 g, egg mass at 51 g/day, and FCR at 1.95 over the 8-week trial (Table 4). Compared with the control group, no differences were observed in performance parameters among hens fed diets containing varying levels of SM or SM-based diets with different levels of YB, except for egg mass (p<0.05). The lowest egg mass was observed in hens fed the diet containing 15% SM and 6% YB; however, this was not different from the other SM-based diets with varying YB levels, except for the 10% SM-9% YB and 15% SM-3% YB diets.

**Figure 2:**
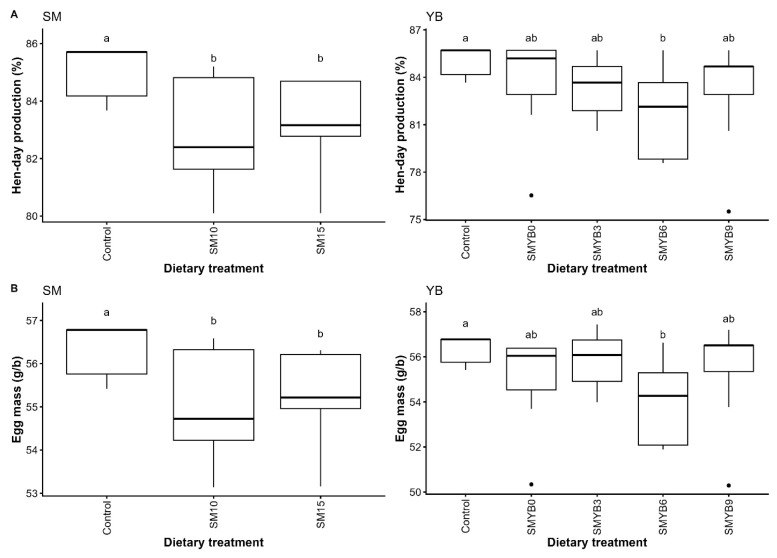
Main effects of varying levels of shrimp meal and yam bean on hen-day egg production (A) and egg mass (B) in laying hens. Data represent the mean±SD (n = 10). ^a,b^ Different superscript letters indicate significant differences at p<0.05. SM, shrimp meal; YB, yam bean; SD, standard deviation; (g/b), unit expressed as grams per bird.

Except egg mass, no interaction between SM and YB was detected for any of the measured parameters; therefore, only the main effects were considered. Both SM and YB affected HDEP and egg mass compared to the control group (p<0.05; Figure 2 and Supplement 2), but had no effect on egg weight, FCR, or BW change. Inclusion of 10% or more SM decreased both HDEP and egg mass compared to the 0% SM diet (p< 0.05). Excluding the control, neither HDEP nor egg mass differed among the different YB levels. Although the feed costs for hens fed diets containing SM and YB were slightly higher than those of the control group, there was no difference in feed cost per kilogram of egg production compared with the control diet, as illustrated in Table 4.

#### Egg quality and nutrient composition

**Figure 3:**
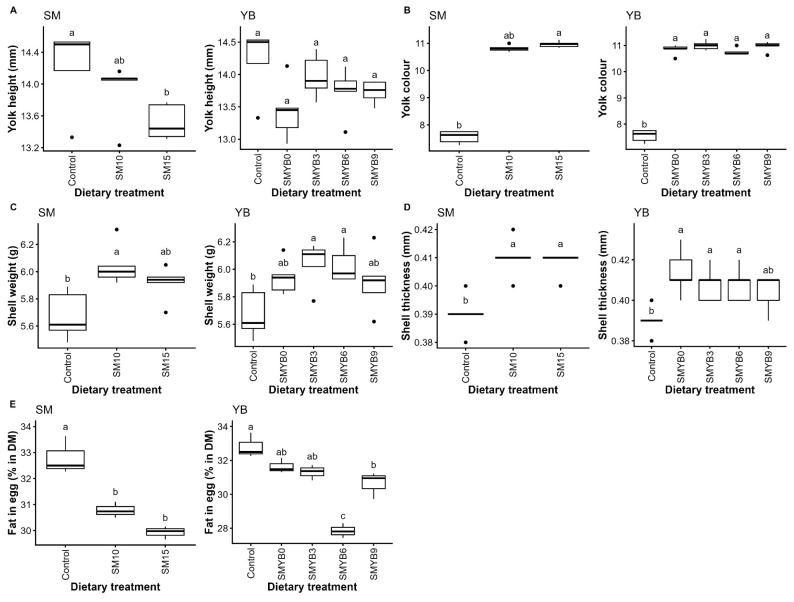
Main effects of varying levels of shrimp meal and yam bean on egg quality and nutrient composition, including yolk height (A), yolk color assessed using the Roche Yolk Color Fan (scale 1–16) (B), shell weight (C), shell thickness (D), and egg fat content (E), in laying hens. Data represent the mean±SD (n = 5). ^a–c^ Different superscript letters indicate significant differences at p<0.05. SM, shrimp meal; YB, yam bean; SD, standard deviation.

No differences were observed among dietary groups receiving various combinations of SM and YB, compared to the control group, except for yolk color (p<0.05) and shell thickness (p<0.05; Table 5). Hens fed SM-based diets with increasing levels of SM exhibited darker yolk color compared to the control (p<0.05). A similar trend of improvement was also observed in shell thickness. When analyzed independently, the addition of SM increased yolk color, shell weight, and shell thickness, but decreased yolk height in a dose-dependent manner (p<0.05; Figure 3 and Supplement 3). However, no differences were observed among SM inclusion levels. Compared to the control, differences were observed in the SM-based diets combined with YB (p<0.05); however, when the control was excluded, increasing YB levels in SM-based diets had no effect on any egg quality parameters.

Regarding egg nutrient composition, varying levels of SM and YB did not affect egg protein content. However, SM-based diets combined with YB significantly reduced egg fat compared to the control (p<0.05). The greatest reductions were observed in the groups receiving 10% SM and 15% SM with 6% YB, where egg fat content was decreased from the control average of 32.8% to 28.0% and 27.8%, respectively. When the main effect was analyzed separately, only egg fat content was affected by SM and YB inclusion compared to the control (p<0.05; Figure 3 and Supplement 3). The SM inclusion resulted in an overall fat reduction of approximately 7.5%, with no differences among SM levels. Egg fat content did not differ among the various YB levels in SM-based diets when the control was excluded; however, the addition of 6% YB resulted in the greatest reduction in egg fat content.

#### Nutrient digestibility and nitrogen balance

**Figure 4:**
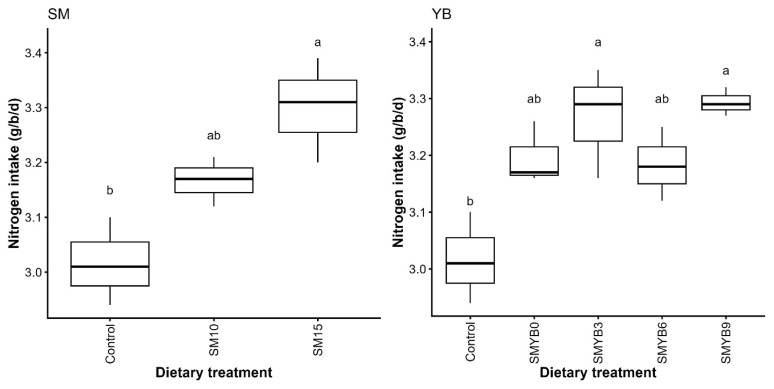
Main effects of varying levels of shrimp meal (SM) and yam bean (YB) on nitrogen (N) intake in laying hens. Data represent the mean ± SD (n = 3). ^a–b^ Different superscript letters indicate significant differences at p<0.05. (g/b/d), unit expressed as grams per bird per day.

The results of nutrient digestibility and N balance are presented in Table 6. Among all parameters measured, a difference was observed only in N retained in eggs (p<0.05). The highest N retention in eggs was found in hens fed the diet containing 10% SM and 9% YB although the difference was not significant compared to the control. When analyzing the main effects independently, N intake was the only parameter affected by both SM and YB inclusion compared to the control (p<0.05; Figure 4 and Supplement 4). N intake increased in a dose-dependent manner with higher levels of SM. However, excluding the control, no differences were observed among the different levels of the YB factor.

## DISCUSSION

### Experiment 1: *in vitro* study

The analysis of chemical composition and *in vitro* digestibility of feed ingredients provides valuable insights into their nutritional suitability for poultry diets. In the present study, SM exhibited high levels of CP and substantial ash content, indicating its potential as a protein-rich supplement that may also supply organic minerals. These characteristics suggest that SM could serve as a promising alternative protein source to soybean meal, with the added benefit of reducing the need for inorganic supplements in laying hen diets. However, the presence of chitin, which makes up approximately 14% of the SM composition, likely contributed to its relatively low IVDMD value of around 48%. Despite some protein in SM being partially enclosed within indigestible chitin, the IVCPD value was still notably high at approximately 74%, suggesting that a significant portion of protein remained accessible. A similar observation was reported by Rahman and Koh [27]. Nevertheless, the protein digestibility of SM was approximately 11% lower than that of soybean meal, which has been reported to be 85% in laying hens [28]. This difference should be taken into account when considering SM as a replacement protein source for soybean meal.

In contrast, YB exhibited a markedly different nutritional profile. Comprising primarily NFE (~88%) and possessing a gross energy value of approximately 3,603 kcal/kg, YB shows its potential as an alternative energy source to conventional ingredients such as maize, which has a metabolizable energy (ME) value of 3,350 kcal/kg according to the NRC [21]. In addition, YB exhibited a higher IVDMD (72.3%) but a lower IVCPD (40.5%) compared to SM. The high IVDMD value suggests efficient digestibility of the carbohydrate-rich fraction (with NFE approximately 85.2%) in YB, supporting its suitability in meeting the energy demands of laying hens. However, the low IVCPD of YB highlights its limited protein value.

To date, limited research has investigated the use of YB as an alternative energy source in laying hen diets. Accordingly, this study aimed to evaluate the appropriate inclusion level of YB in combination with 10% SM in the diets of laying hens. Notably, although SM had lower protein digestibility than soybean meal, the diet containing 10% SM did not differ significantly in *in vitro* digestibility compared to the control diet (based on maize and soybean meal), indicating that this level of SM inclusion is suitable and did not impair nutrient digestibility. Additionally, increasing levels of YB in SM-based diet had no adverse effect on either IVDMD or IVCPD. These findings clearly demonstrate that the combination of SM (up to 10%) and YB (up to 9%) can be used as alternative feed ingredients in laying hen diets without negatively affecting nutrient digestibility.

### Experiment 2: *in vivo* study

The *in vivo* study demonstrated that diets containing 10% or 15% SM combined with various YB levels showed no differences in term of laying performance from the control diet except for the egg mass. Notably, the combination of SM and 6% YB led to a reduction in egg mass, which may be attributed to the individual effects of each component when considered separately. When evaluated independently, increasing levels of SM negatively affected HDEP and egg mass, even at 10% inclusion. These findings were consistent with previous studies by Rahman and Koh [11] and Sangkaew et al [29], which reported that overall laying performance declined when SM level exceeded 10%. The observed reduction in HDEP and egg mass may be due to chitin, an antinutritional factor found in SM [17], which was present at similar levels (approximately 14%) across studies. Chitin, a structural polysaccharide composed of N-acetylglucosamine, is largely indigestible for poultry and can suppress the digestibility of other nutrients [17]. Mechanistically, chitin can form complexes with dietary proteins, reducing their accessibility to proteolytic enzymes and thereby hindering protein hydrolysis. As an insoluble dietary fiber has very low viscosity, chitin may also decrease transit time in the small intestine, shortening the time available for absorption and ultimately lowering overall digestibility [30]. Consequently, the presence of chitin in the diet reduces nutrient utilization efficiency and increases the digestive workload required to process other nutrients, diverting energy away from productive functions such as egg production.

Main effect analysis also revealed a similar trend for YB, where increasing inclusion levels led to reductions in HDEP and egg mass compared to the control, with a significant effect observed at 6% YB. These findings suggest that the 6% YB level was the primary contributor to the reduced egg mass observed in the groups receiving 10% and 15% SM combined with 6% YB. The mechanism underlying this negative impact of YB remains unclear, as YB is generally considered low in antinutritional content [4]. However, Shi et al [31] reported that YB (*P. erosus*) starch contains a high proportion of resistant starch (approximately 65% to 70%) compared to only 11.8% in corn starch [32], which may reduce digestibility and energy utilization in poultry [31]. Due to its high crystallinity and compact granule structure, resistant starch is resistant to hydrolysis by pancreatic amylase [33]. The reduced starch breakdown limits glucose availability for absorption, resulting in lower ME and overall feed efficiency. Although undigested resistant starch can reach the ceca and undergo fermentation into short-chain fatty acids (such as acetate, propionate, and butyrate), which can support gut health [33], excessive fermentation may shift energy utilization away from egg production. This could partially explain the observed decline in HDEP and egg mass in laying hens fed YB-containing diets. Despite the fact that SM and YB independently exert negative effects on HDEP, their combined inclusion did not result in significant differences from the control. Therefore, SM and YB still show potential as eco-friendly protein and energy alternatives, respectively. Inclusion at moderate levels is recommended for optimal results. Additionally, the cost of feeding per kilogram of egg production did not differ significantly among laying hens fed diets containing various levels of SM and YB compared with those fed the control diet, indicating the economic feasibility of incorporating SM and YB as locally available alternative ingredients in place of conventional feed components.

Egg quality is a key determinant of production efficiency and profitability. It is well known that both egg quality and its nutrient composition depend on the hens’ daily nutrient intake. In this study, the combination of varying levels of SM and YB had no adverse effects on egg quality or nutrient composition, supporting their potential as local alternatives to conventional feed ingredients such as maize and soybean meal. Although improvements in yolk color and shell thickness were observed in diets containing both SM and YB, independent analyses indicated that SM was the main contributor. The SM enhanced yolk color and shell quality compared to the control, while YB alone had minimal effect, as no differences were observed among the different YB inclusion levels. The SM-containing diets without YB still outperformed others. The improvement in yolk pigmentation is likely attributable to the astaxanthin content in SM. Honda et al [16] reported yolk color scores above 14 with 8 mg/kg astaxanthin from *Paracoccus carotinifaciens*. Similar findings were also observed in other studies [9,11,29]. Additionally, SM had a strong positive effect on shell quality, as evidenced by increased shell weight and thickness. Similarly, consistent with the findings reported by Rahman and Koh [11] and Sangkaew et al [29]. The enhanced shell quality may be attributed to SM’s high organic mineral content, as indicated in its ash content of approximately 25%. Rahman and Koh [11] also reported a positive correlation between calcium levels in SM and eggshell strength. These findings suggest that the organic minerals in SM may be more bioavailable than inorganic calcium carbonate typically used in poultry diets, despite equivalent calcium levels across diets.

Egg fat content significantly decreased with SM inclusion. This may be due to chitin in SM, which binds anionic bile salts, limiting their ability to displace emulsifiers and reducing lipid surface area for lipase action [34]. This, in turn, decreases fat absorption and promotes fecal excretion. This finding aligns with Nuraini et al [35], who reported reduced yolk fat in quails fed chitin-rich Hong Kong caterpillars. The reduced egg fat observed in SM-fed hens may also relate to the decreased yolk height, as fat is primarily deposited in the yolk rather than the albumen, and yolk weights did not differ among groups. Although 6% YB significantly reduced egg fat content, this effect was not observed at other YB levels and was not consistent across SM levels, suggesting that the reduction may not be attributable to YB itself but could be due to other factors such as individual variation or hen condition in those treatment groups.

The combination of varying levels of SM and YB in the diets had no adverse effects on nutrient digestibility or N retention in laying hens, consistent with the *in vitro* digestibility findings. This further confirms that the inclusion of SM and YB did not impair nutrient utilization. Overall, the results suggest that the combined use of these eco-friendly, locally available ingredients is more effective than using each one individually. When the main factor was considered independently, increasing SM levels led to higher N intake, particularly at 15% SM. This was attributed to the increased FI at the 15% level compared to the control, as N intake is directly related to the amount of feed consumed. Although a similar trend was noted with YB compared to the control, the dose-dependent increase in N intake was inconsistent, and higher values were also observed in SM-based diets without YB. This suggests that the increase in N intake is primarily driven by SM inclusion rather than by YB.

## CONCLUSION

The findings of this study highlight that SM and YB are promising alternative protein and energy sources, respectively, for laying hen diets. Their inclusion promotes sustainable poultry feed production by utilizing processing by-products and locally underused crops. SM is rich in protein, while YB provides high levels of NFE and gross energy comparable to maize. *In vitro* assessments showed that diets containing 10% SM with up to 9% YB had no adverse effects on nutrient digestibility. Consistent results were observed *in vivo*, where SM-based diets with varying YB inclusion levels did not negatively impact laying performance, egg quality, egg nutrient composition, nutrient digestibility, or N retention. Notably, the combination of SM and YB at inclusion levels of up to 15% and 9%, respectively, yielded optimal results without compromising productivity or nutrient utilization.

## Figures and Tables

**Table 1:** Chemical compositions and *in vitro* digestibility of shrimp meal and yam bean

Items^1)^	Shrimp meal	Yam bean
Crude protein (%)	47.69±0.15	6.13±0.09
Ether extract (%)	6.78±0.32	0.44±0.03
Crude fiber (%)	11.01±0.69	3.14±0.01
Ash (%)	24.87±0.41	1.96±0.11
Nitrogen-free extract (%)	10.24±0.85	88.07±0.50
Gross energy (kcal/kg)	3,803.52±19.60	3,602.65±7.69
Chitin (%)	14.07±0.39	-
*In vitro* digestibility		
IVDMD (%)	47.78±1.59	72.27±4.02
IVCPD (%)	73.51±0.51	40.54±4.41

1) The values of each parameter represent the mean values±SD of triplicate analyses (in dry matter).

IVDMD, *in vitro* dry matter digestibility; IVCPD, in vitro crude protein digestibility; SD, standard deviation.

**Table 2:** Composition and nutrient level of the experimental diets for *in vitro* experiment

Ingredients (%) (as-fed basis)	Dietary groups				

	Control	SM10	SM10-YB3	SM10-YB6	SM10-YB9
Maize	61.0	60.1	56.1	52.1	48.1
Soybean meal	27.2	18.3	18.5	18.7	18.9
Yam bean (YB)	0.0	0.0	3.0	6.0	9.0
Shrimp meal (SM)	0.0	10.0	10.0	10.0	10.0
Palm oil	0.4	0.5	1.3	2.1	2.9
DL-methionine	0.2	0.1	0.1	0.1	0.1
L-lysine	0.2	0.3	0.3	0.3	0.3
Vitamin-mineral premix	0.3	0.3	0.3	0.3	0.3
Dicalcium phosphate	1.6	1.1	1.1	1.1	1.1
Calcium carbonate	8.7	9.1	9.1	9.1	9.1
Salt	0.5	0.4	0.4	0.4	0.4
Nutritional value (%)^1)^					
Crude protein	19.03	19.99	19.90	19.96	19.41
Crude fiber	2.78	3.70	4.10	4.50	4.90

All diets were formulated to meet the nutrient requirement of laying hens, as recommended by NRC [21].

Vitamin-mineral premixes provided the following per kilogram of premix: vitamin A, 4,000,000 IU; vitamin D3, 800,000 IU; vitamin E, 4,800 IU; vitamin K, 600 mg; thiamin, 600 mg; riboflavin, 1,600 mg; pyridoxine, 1,600 mg; vit 12, 7 mg; pantothenic acid, 4,000 mg; folic acid, 170 mg; nicotinic acid, 8,800 mg; biotin, 40 mg; copper, 3,200 mg; manganese, 28,000 mg; zinc, 20,000 mg; iron, 24,000 mg; iodine, 280 mg; cobalt, 200 mg; selenium, 40 mg; antioxidants, 500 mg.

The metabolizable energy in all diets was calculated to be approximately 2,752 to 2,754 kcal/kg, with calcium at around 3.73% and phosphorus at approximately 0.38%.

1) The values of each parameter represent the mean values of triplicate analyses (in dry mater).

**Table 3:** Composition and nutrient level of the experimental diets for the *in vivo* experiment

Ingredients (%) (as-fed basis)	Control	10% SM				15% SM			
	
		0% YB	3% YB	6% YB	9% YB	0% YB	3% YB	6% YB	9% YB
Maize	61.0	60.1	56.1	52.1	48.1	61.2	56.9	52.8	48.6
Soybean meal	27.2	18.3	18.5	18.7	18.9	12.5	12.9	13.3	13.6
Yam bean (YB)	0.0	0.0	3.0	6.0	9.0	0.0	3.0	6.0	9.0
Shrimp meal (SM)	0.0	10.0	10.0	10.0	10.0	15.0	15.0	15.0	15.0
Palm oil	0.4	0.5	1.3	2.1	2.9	0.5	1.3	2.1	2.9
DL-methionine	0.2	0.1	0.1	0.1	0.1	0.1	0.1	0.1	0.1
L-lysine	0.2	0.3	0.3	0.3	0.3	0.3	0.3	0.3	0.3
Vitamin-mineral premix	0.3	0.3	0.3	0.3	0.3	0.3	0.3	0.3	0.3
Dicalcium phosphate	1.6	1.1	1.1	1.1	1.1	0.8	0.8	0.8	0.8
Calcium carbonate	8.7	9.1	9.1	9.1	9.1	9.3	9.3	9.3	9.3
Salt	0.5	0.4	0.4	0.4	0.4	0.3	0.3	0.3	0.3
Nutritional value (%)^1)^									
Crude protein	18.69	19.39	19.42	19.68	19.45	19.79	20.11	19.95	19.77
Crude fiber	2.78	3.14	3.14	3.15	3.16	3.28	3.29	3.30	3.31
Ether extract	2.94	3.77	4.10	4.95	5.22	3.96	3.98	5.24	5.61
Feed cost (baht/kg diet)^2)^	15.31	15.72	15.83	15.94	16.06	15.95	16.05	16.15	16.26

All diets were formulated to meet the nutrient requirement of laying hens, as recommended by ISA Brown commercial management guide [23].

Vit-mineral premixes provided the following per kilogram of premix: vitamin A, 4,000,000 IU; vitamin D_3_, 800,000 IU; vitamin E, 4,800 IU; vitamin K, 600 mg; thiamin, 600 mg; riboflavin, 1,600 mg; pyridoxine, 1,600 mg; vit 12, 7 mg; pantothenic acid, 4,000 mg; folic acid, 170 mg; nicotinic acid, 8,800 mg; biotin, 40 mg; copper, 3,200 mg; manganese, 28,000 mg; zinc, 20,000 mg; iron, 24,000 mg; iodine, 280 mg; cobalt, 200 mg; selenium, 40 mg; antioxidants, 500 mg.

The metabolizable energy in all diets was calculated to be approximately 2,752 to 2,754 kcal/kg, with calcium at around 3.73% and phosphorus at approximately 0.38%.

1) The values of each parameter represent the mean values of triplicate analyses (in dry mater).

2) Calculated value.

**Table 4:** Effects of shrimp meal and yam bean inclusion on the laying performance of laying hens

Dietary groups		HDEP (%)	FI (g/b/d)	Egg weight (g)	Egg mass (g/b/d)	FCR	BW change (g/b/8 weeks)	Feed cost (bath/1 kg egg)
Control		85.1±1.0	109.9±3.3	59.6±2.6	56.4±0.6^ab^	1.95±0.07	179.2±59.8	29.9±1.07
SM (%)	YB (%)							
10	0	83.9±3.1	109.9±4.3	59.1±3.9	54.8±2.2^ac^	1.98±0.13	157.4±69.0	31.1±2.00
	3	83.5±2.8	108.0±4.8	59.3±1.3	54.8±2.0^abc^	1.97±0.10	176.3±69.5	31.1±1.66
	6	82.0±4.3	107.7±2.5	59.7±2.7	53.8±2.9^abc^	1.98±0.09	166.9±181.3	31.5±1.50
	9	81.8±6.3	112.3±5.4	61.0±0.9	55.5±4.6^ab^	2.04±0.22	192.1±76.0	32.7±3.53
15	0	83.7±3.8	109.3±5.0	59.3±1.5	54.8±2.8^bc^	1.99±0.12	187.6±125.5	31.7±1.84
	3	83.3±2.3	110.5±4.9	61.3±1.8	56.7±2.8^b^	1.95±0.11	152.8±115.7	31.3±1.85
	6	81.4±3.7	109.8±7.0	59.2±1.0	53.0±2.4^c^	2.04±0.18	130.1±164.6	33.0±2.90
	9	84.5±2.0	110.3±2.3	59.2±1.4	56.0±0.6^abc^	1.99±0.07	191.5±128.0	32.3±1.10

The values represent the mean±standard deviation of ten replicates per treatment.

a–c Different letters indicate significant differences among treatments at p<0.05.

HDEP, hen-day egg production; FI, feed intake; FCR, feed conversion ratio; BW, body weight; SM, shrimp meal; YB, yam bean.

**Table 5:** Effects of shrimp meal and yam bean inclusion on the egg quality and egg nutrient composition of laying hens

Dietary groups		Egg quality^1)^							Nutrient composition^2)^	

		Haugh units	Albumin height (mm)	Yolk height (mm)	Yolk weight (g)	Yolk color	Shell weight (g)	Shell thickness (mm)	Egg protein (%)	Egg fat (%)
Control		105.8±6.2	12.1±1.8	14.2±0.5	13.9±0.6	7.6±0.2^d^	5.7±0.2	0.39±0.008^b^	51.5±1.40	32.8±0.73^a^
SM (%)	YB (%)									
10	0	104.9±4.2	11.4±1.1	13.2±0.7	13.7±0.5	10.8±0.2^bc^	6.0±0.2	0.41±0.009^ab^	52.5±0.77	31.9±0.68^ab^
	3	105.6±6.1	11.7±1.4	14.2±0.9	14.0±0.3	11.3±0.3^a^	6.1±0.2	0.41±0.015^ab^	52.8±1.84	31.6±1.49^ab^
	6	106.3±3.7	11.8±0.9	14.0±0.8	14.1±1.4	10.5±0.3^c^	6.1±0.2	0.41±0.008^ab^	54.1±1.91	28.0±1.08^c^
	9	105.5±6.2	12.1±1.5	14.2±0.3	14.3±0.3	10.8±0.1^abc^	6.0±0.2	0.41±0.010^ab^	52.7±0.71	31.7±0.75^ab^
15	0	108.7±3.0	12.4±0.8	13.7±0.5	13.8±1.0	10.9±0.2^abc^	5.9±0.1	0.42±0.014^a^	51.1±2.58	31.4±1.00^ab^
	3	104.6±3.9	11.4±1.0	13.7±0.4	14.3±0.7	10.7±0.1^bc^	6.0±0.3	0.41±0.009^ab^	51.5±1.02	31.0±1.04^ab^
	6	108.0±3.0	12.2±0.8	13.4±0.4	13.3±0.6	11.1±0.2^ab^	6.0±0.2	0.40±0.009^ab^	52.6±1.19	27.7±0.21^c^
	9	109.5±1.3	12.6±0.3	13.3±0.2	13.9±0.5	11.1±0.3^ab^	5.8±0.3	0.40±0.014^ab^	53.3±1.26	29.6±0.92^bc^

1) The values represent the mean±standard deviation of five replicates per treatment.

2) The values of each parameter represent the mean values of triplicate analyses (in dry mater).

a–c Different letters indicate significant differences among treatments at p<0.05.

SM, shrimp meal; YB, yam bean.

**Table 6:** Effects of shrimp meal and yam bean inclusion on the nitrogen balance and nutrient digestibility in laying hens

Dietary groups		N intake (g/b/d)	N excreta (g/b/d)	Retained N (g/b/d)			Dry matter digestibility (%)	Fat digestibility (%)

				Total^1)^	Egg^2)^	Body^3)^		
Control		3.02±0.08	1.39±0.20	1.63±0.25	1.10±0.00^ab^	0.53±0.25	77.4±3.12	85.9±8.41
SM (%)	YB (%)							
10	0	3.09±0.17	1.45±0.26	1.63±0.27	1.09±0.00^ab^	0.54±0.27	77.8±3.01	86.8±3.74
	3	3.19±0.10	1.52±0.14	1.68±0.13	1.07±0.00^ab^	0.61±0.13	76.3±2.89	86.5±3.53
	6	3.07±0.20	1.51±0.23	1.56±0.03	1.07±0.04^ab^	0.49±0.07	75.4±1.58	86.0±3.82
	9	3.32±0.07	1.47±0.11	1.85±0.17	1.12±0.02^a^	0.73±0.16	77.4±1.22	86.0±3.22
15	0	3.31±0.14	1.16±0.34	2.15±0.47	1.06±0.06^b^	1.09±0.42	80.5±4.56	86.1±1.88
	3	3.34±0.11	1.44±0.20	1.90±0.47	1.10±0.00^ab^	0.79±0.24	77.3±2.32	84.4±2.91
	6	3.30±0.29	1.50±0.23	1.80±0.50	1.06±0.04^b^	0.74±0.53	74.8±5.96	85.7±5.88
	9	3.27±0.06	1.55±0.15	1.76±0.20	1.08±0.02^ab^	0.67±0.21	75.5±2.12	75.5±8.75

The values of each parameter represent the mean values of triplicate analyses (in dry mater).

1) Total N retention was determined by subtracting N excretion from N intake.

2) Retained N content in eggs was calculated by multiplying the egg mass by 1.936, following the method described by Roberts et al [24].

3) Body N retention was calculated as the difference between total retained N and the N retained in eggs [24].

a,b Different letters indicate significant differences among treatments at p<0.05.

N, nitrogen; SM, shrimp meal; YB, yam bean.

## Data Availability

Upon reasonable request, the datasets of this study can be available from the corresponding author.

## SUPPLEMENTARY MATERIAL

**Supplement 1**. Effects of shrimp meal and yam bean inclusion on *in vitro* digestibility of dry matter and crude protein.

**Supplement 2**. Main effects of varying levels of shrimp meal and yam bean on the laying performance of laying hens.

**Supplement 3**. Main effects of varying levels of shrimp meal and yam bean on egg quality and nutrient composition of laying hens.

**Supplement 4**. Main effects of varying levels of shrimp meal (SM) and yam bean (YB) on nitrogen (N) retention in laying hens.
