# Supplementary material for: Nutritional and performance effects of shrimp meal and yam bean as sustainable ingredients in laying hen diets
Source: Anim Biosci. 2025 Dec 18;39(5):250559. doi: 10.5713/ab.250559 (PMC13175071; doi:10.5713/ab.250559)
Supplement: Supplementary file 1 [file ab-250559-Supplement-1.pdf]

Supplement 1. Effects of shrimp meal and yam bean inclusion on *in vitro* digestibility of dry matter and crude protein.

**Dietary groups**[1]	**IVDMD (% on DM basis)**[2]	**IVCPD (% on DM basis)**[3]
Control	62.91±3.19	64.90±3.73
SM10YB0	58.89±1.34	64.45±1.03
SM10YB3	60.59±2.56	68.50±1.73
SM10YB6	59.30±1.51	69.67±1.21
SM10YB9	59.11±0.88	69.12±1.73

[1] SM = shrimp meal; YB = yam bean

[2] IVDMD = *in vitro* dry matter digestibility. The values represent the mean values ± standard deviation of triplicate analyses (in dry matter).

[3] IVCPD = *in vitro* crude protein digestibility. The values represent the mean values ± standard deviation of triplicate analyses (in dry matter).
