# Supplementary material for: Nutritional and performance effects of shrimp meal and yam bean as sustainable ingredients in laying hen diets
Source: Anim Biosci. 2025 Dec 18;39(5):250559. doi: 10.5713/ab.250559 (PMC13175071; doi:10.5713/ab.250559)
Supplement: Supplementary file 2 [file ab-250559-Supplement-2.pdf]

Supplement 2. Main effects of varying levels of shr imp meal and yam bean on the laying performance of laying hens[1,2].

**Dietary groups**	**HDEP (%)**	**FI (g/b/d)**	**Egg mass (g/b/d)**	**FCR**	**BW change (g/b)**
Control	85.10±0.99	109.90±3.27	56.40±0.65	1.95±0.07	179.20±59.78
SM10	82.80±1.92	109.20±1.95	55.00±1.28	1.99±0.04	174.00±58.21
SM15	83.20±1.60	110.00±3.31	55.20±1.07	2.00±0.08	166.98±41.15
YB0	83.80±2.94	109.10±2.59	55.10±1.94	1.98±0.08	172.50±87.63
YB3	83.40±1.67	109.30±4.43	55.90±1.12	1.96±0.08	166.21±76.34
YB6	81.70±2.82	108.80±4.35	54.00±1.87	2.01±0.10	148.50±129.89
YB9	83.20±3.05	111.30±3.53	55.50±2.07	2.01±0.11	191.80±80.00

[1] SM = shrimp meal; YB = yam bean; HDEP = hen-day egg production; FI = feed intake; BW = body weight; FCR = feed conversion ratio.

[2] The values represent the mean ± standard deviation of ten replicates per treatment.
