# Supplementary material for: Nutritional and performance effects of shrimp meal and yam bean as sustainable ingredients in laying hen diets
Source: Anim Biosci. 2025 Dec 18;39(5):250559. doi: 10.5713/ab.250559 (PMC13175071; doi:10.5713/ab.250559)
Supplement: Supplementary file 3 [file ab-250559-Supplement-3.pdf]

Supplement 3. Main effects of varying levels of shrimp meal and yam bean on egg quality and nutrient composition of laying hens [1].

Dietary groups	Egg quality[2]							Nutrient composition[3]	
	Haugh units	Albumin height, mm	Yolk Height, mm	Yolk weight, g	Yolk color	Shell weight, g	Shell thickness, mm	Egg protein, %	Egg fat, %
Control	105.80±6.25	12.06±1.80	14.21±0.52	13.86±0.55	7.55±0.23	5.68±0.17	0.39±0.01	51.50±1.40	32.80±0.73
SM10	105.50±3.86	11.74±0.98	13.91±0.39	14.05±0.39	10.82±0.12	6.05±0.15	0.41±0.01	53.00±0.24	30.80±0.31
SM15	107.70±0.75	12.17±0.22	13.52±0.22	13.82±0.40	10.96±0.11	5.92±0.13	0.41±0.00	52.10±0.56	30.20±0.21
YB0	106.80±2.88	11.87±0.82	13.44±0.45	13.80±0.48	10.84±0.20	5.94±0.12	0.42±0.01	51.80±1.03	31.60±0.44
YB3	105.10±2.91	11.58±0.73	13.97±0.33	14.19±0.46	11.00±0.17	6.04±0.16	0.41±0.01	52.10±0.87	31.30±0.46
YB6	107.10±3.02	12.00±0.81	13.73±0.38	13.67±0.64	10.76±0.14	6.03±0.13	0.41±0.01	53.40±0.38	28.10±0.89
YB9	107.50±2.65	12.37±0.66	13.73±0.17	14.10±0.28	10.96±0.20	5.91±0.22	0.40±0.01	53.00±0.47	30.60±0.80

[1] SM = shrimp meal; YB = yam bean.

[2] The values represent the mean ± standard deviation of five replicates per treatment.

[3] The values of each parameter represent the mean values of triplicate analyses (in dry matter).
