# Supplementary material for: Nutritional and performance effects of shrimp meal and yam bean as sustainable ingredients in laying hen diets
Source: Anim Biosci. 2025 Dec 18;39(5):250559. doi: 10.5713/ab.250559 (PMC13175071; doi:10.5713/ab.250559)
Supplement: Supplementary file 4 [file ab-250559-Supplement-4.pdf]

Supplement 4. Main effects of varying levels of shrimp meal (SM) and yam bean (YB) on nitrogen (N) retention in laying hens[1,2].

Dietary groups	N intake, g/b/d	N excreta, g/b/d	Retained N, g/b/d			Dry matter digestibility, %	Fat digestibility, %
			Total[3]	Egg[4]	Body[5]		
Control	3.02±0.08	1.39±0.20	1.63±0.25	1.10±0.00	0.53±0.25	77.43±3.12	85.91±8.41
SM10	3.17±0.05	1.49±0.11	1.68±0.07	1.08±0.01	0.59±0.07	76.73±0.91	86.34±0.57
SM15	3.30±0.09	1.41±0.02	1.90±0.11	1.08±0.01	0.82±0.12	77.03±1.13	82.91±1.71
YB0	3.20±0.05	1.31±0.14	1.89±0.12	1.07±0.03	0.82±0.10	79.15±1.84	86.42±1.49
YB3	3.27±0.10	1.48±0.10	1.79±0.06	1.09±0.00	0.70±0.06	76.77±0.70	85.48±2.85
YB6	3.18±0.06	1.51±0.21	1.68±0.26	1.06±0.04	0.61±0.29	75.13±3.66	85.83±1.03
YB9	3.29±0.03	1.51±0.06	1.80±0.07	1.10±0.02	0.70±0.07	76.49±1.24	80.76±5.68

[1] SM = shrimp meal; YB = yam bean.

[2] The values of each parameter represent the mean values of triplicate analyses (in dry matter).

[3] Total nitrogen retention was determined by subtracting nitrogen excretion from nitrogen intake.

[4] Retained nitrogen content in eggs was calculated by multiplying the egg mass by 1.936, following the method described by Roberts et al. [24].

[5] Body nitrogen retention was calculated as the difference between total retained nitrogen and the nitrogen retained in eggs [24].
